# 190. Epidemiology of COVID-19 Breakthrough Infections in Dallas County, Texas, 2021

**DOI:** 10.1093/ofid/ofab466.190

**Published:** 2021-12-04

**Authors:** Suzanne Wada, Jared Wiegand, Mary Markarian, Victoria Hung, Christina Zhu, Megin Parayil, Kyoo Shim, Jose Serrano, Wendy Chung

**Affiliations:** 1 Dallas County Health and Human Services, Dallas, Texas; 2 Dallas County Department of Health and Human Services, Dallas, TX

## Abstract

**Background:**

From March 2020 through May 2021, Dallas County reported a total of 304,056 cases of COVID-19, including 4,073 deaths. During the month of December 2020, a post-holiday surge of cases led to peak daily average case rates of over 50 cases per 100,000. COVID-19 cases and deaths have since declined substantially following the rollout of COVID-19 vaccine delivery. As of June 8, 2021, about 1,831,588 Dallas County residents have received at least one COVID-19 vaccine dose and 910,067 are fully vaccinated. Recent county integration of immunization and case databases enabled identification and analysis of COVID-19 breakthrough infections.

**Methods:**

A COVID-19 breakthrough infection was defined as a positive test (PCR or antigen) collected from an individual ≥ 14 days after receiving the full series of an FDA-authorized COVID-19 vaccine. Nationally, 10,262 vaccine breakthrough infections had been reported from 46 US states and territories, through April 2021. Vaccine breakthrough cases were reviewed and medical records abstracted to collect demographic information, clinical characteristics, and medical conditions. Data analysis was performed using R, version 4.0.2 (2020).

**Results:**

Of the 700 vaccine breakthrough cases reported in Dallas County residents as of June 8, 2021, 304 (43%) were male and 396 (57%) female, with an average age of 53 years. The majority of the vaccine breakthrough cases were White (42%); 25% were Hispanic/Latino; and 20% were Black. Almost all breakthrough cases were confirmed with PCR testing, with 451 (64%) cases receiving the Pfizer vaccine. Of breakthrough cases, 49% were symptomatic; 52% (358) had underlying conditions including: tobacco use, obesity, or immunocompromised state; 68 (10%) were hospitalized; and 11 (1.6%) died. Whole genome sequencing was performed on 51 cases, with 14 (27.5%) variants identified, including: eight B.1.1.7, two B.1.429 and one P.1 variants.

**Conclusion:**

Despite the high levels of vaccine efficacy documented in US vaccine trials, COVID-19 breakthrough infections, though currently uncommon, do occur and are important to investigate. Ongoing close public health surveillance of variants is needed to discern changes in patterns of vaccine efficacy and characteristics of populations at greatest risk of severe disease from COVID-19.

**Disclosures:**

**All Authors**: No reported disclosures

